# An Unusual Bacterial‐Fungal Splenic Abscess in an Immunocompetent Host: *Salmonella Paratyphi A* and *Candida tropicalis* Co‐Infection

**DOI:** 10.1155/crdi/3533752

**Published:** 2026-10-05

**Authors:** Kapil Chahal, Neha Rastogi, Swati Bhayana, Amit Javed, Rinkesh Kumar Bansal, Zubin Dev Sharma, Saurabh Kumar, Narola Yanger, Neha Lal, Shrinidhi Nathany, Vikas Dua, Rahul Bhargava

**Affiliations:** ^1^ Department of Infectious Diseases Fortis Memorial Research Institute & Hospital Gurugram India; ^2^ Department of Haematology, Paediatric Oncology Fortis Memorial Research Institute & Hospital Gurugram India; ^3^ Department of Gastroenterology and Hepatobiliary Sciences Fortis Memorial Research Institute & Hospital Gurugram India; ^4^ Department of Interventional Radiology Fortis Memorial Research Institute & Hospital Gurugram India jsd.js.cn; ^5^ Department of Microbiology Fortis Memorial Research Institute & Hospital Gurugram India; ^6^ Department of Haematology Fortis Memorial Research Institute & Hospital Gurugram India

## Abstract

Splenic abscess is a rare but potentially life‐threatening condition, most frequently seen in immunocompromised individuals or those with significant underlying comorbidities. Monomicrobial bacterial infections predominate, whereas fungal or mixed infections are uncommon, particularly in immunocompetent hosts. Concurrent bacterial and fungal infection of the spleen is exceedingly rare. We report the case of a 30‐year‐old immunocompetent Indian male who presented with high‐grade fever, left upper quadrant abdominal pain, and gastrointestinal symptoms. Imaging demonstrated a multiseptated cystic lesion in the upper pole of the spleen consistent with abscess formation. Endoscopic ultrasound‐guided fine‐needle aspiration yielded purulent material, and culture revealed dual infection with *Salmonella enterica* serovar Paratyphi A and *Candida tropicalis*. Blood cultures and tuberculosis workup were negative. Despite broad‐spectrum empirical antimicrobial therapy, radiological progression necessitated laparoscopic drainage and debridement, followed by culture‐directed antibacterial therapy for a total of 5 weeks and antifungal therapy for 6 weeks. The patient achieved complete clinical and radiological resolution with preservation of splenic function. This case highlights the importance of maintaining a high index of clinical suspicion for splenic abscess and considering polymicrobial infection, including fungal pathogens, even in immunocompetent patients. Prompt microbiological diagnosis, early multidisciplinary involvement, timely spleen‐sparing source control, and targeted antimicrobial therapy guided by susceptibility testing facilitated successful organ preservation while avoiding splenectomy, ultimately resulting in a favorable clinical outcome.

## 1. Background

Splenic cysts are uncommon, with an estimated incidence of approximately 0.07%–0.13% in the general population [1]. Most remain asymptomatic and are detected incidentally on imaging. However, larger cysts may cause compressive symptoms or develop complications such as hemorrhage, rupture, or secondary infection [1]. Infected splenic cysts can progress to splenic abscess, a rare but potentially life‐threatening condition. Splenic abscess has an autopsy‐reported prevalence of 0.14%–0.7% and a clinical incidence of approximately 0.012–0.056 per 100,000 hospital admissions annually [2]. It is most frequently associated with immunocompromised states and systemic comorbidities, including diabetes mellitus, malignancy, infective endocarditis, trauma, and immunodeficiency. The microbiological spectrum is broad, with *Staphylococcus aureus*, *Streptococcus* species, *Escherichia coli*, and *Mycobacterium tuberculosis* among commonly reported pathogens [3]. *Salmonella enterica* serovars are less frequently implicated, and fungal etiologies are typically observed in immunocompromised hosts. Concurrent bacterial and fungal infection of the spleen is exceedingly rare, particularly in immunocompetent individuals.

We report a case of an infected splenic cyst progressing to splenic abscess caused by dual infection with *Salmonella Paratyphi A* and *Candida tropicalis* in an immunocompetent adult, highlighting the importance of microbiological evaluation and early source control in achieving favorable outcomes while preserving splenic function.

## 2. Case Presentation

A 30‐year‐old previously healthy Indian male presented to the emergency department of our hospital with high‐grade fever associated with chills and rigors for 8–10 days, including continuous nonremitting fever for the preceding 3‐4 days. He also reported progressive left upper quadrant abdominal pain for 5–7 days, nausea with recurrent vomiting, loose stools (7‐8 episodes per day), generalized weakness, and severe lethargy. The patient had experienced two similar self‐limiting febrile episodes in the past year, both resolving with empirical oral medications without further evaluation. Ten days prior to the current admission, he developed persistent high‐grade fever with abdominal pain that did not respond to oral therapy. An ultrasound performed at a local hospital revealed a thin‐walled unilocular splenic cyst with features suggestive of infection or abscess formation. He was initially treated with oral cefuroxime (500 mg twice daily). Because of persistent fever and abdominal pain, therapy was subsequently escalated to intravenous ceftriaxone (2 g twice daily). After 48 h without significant clinical improvement, ceftriaxone was replaced with intravenous meropenem (1 g every 8 h). Despite a further 48 h of meropenem therapy, fever and abdominal pain persisted, with worsening gastrointestinal symptoms, prompting referral to our centre. A chest radiograph obtained at the referring hospital demonstrated haziness in the left lower zone.

On examination at admission, his temperature was 102°F. The patient was hemodynamically stable with a Glasgow Coma Scale score of E4V5M6. Respiratory examination revealed bilateral air entry with mild wheeze and crepitations in the left lower lobe. Abdominal examination demonstrated tenderness in the epigastrium and left hypochondrium. On admission, intravenous meropenem (1 g every 8 h), which had been initiated at the referring hospital, was continued. Empirical coverage was broadened by adding intravenous teicoplanin (400 mg every 12 h for three loading doses, followed by 400 mg once daily) and oral doxycycline (100 mg twice daily) while further diagnostic evaluation was undertaken.

Initial laboratory investigations revealed elevated inflammatory markers, with a procalcitonin level of 3.91 ng/mL and a C‐reactive protein (CRP) level of 210 mg/L. Hemoglobin was 11.5 g/dL, total leukocyte count was 12.64 × 10^9^/L, platelet count was 282 × 10^9^/L, aspartate aminotransferase (AST/SGOT) was 128 U/L, alanine aminotransferase (ALT/SGPT) was 169 U/L, and serum creatinine was 0.89 mg/dL. Paired blood cultures, urine culture, and the Widal test were negative. An extensive tropical infectious disease workup, including tests for malaria, dengue, leptospirosis, scrub typhus, Epstein–Barr virus serology, and *Brucella* antibodies, was also negative. The patient underwent an evaluation for underlying immunodeficiency, including fourth‐generation HIV serology (negative), HbA1c (4.9%), CD4+ T‐cell count (468 cells/µL), and serum immunoglobulin profile (IgG, IgA, and IgM), all of which were within normal limits. There was no history of malignancy, transplantation, chronic liver disease, diabetes mellitus, or chronic corticosteroid or other immunosuppressive therapy. Advanced genetic testing for inborn errors of immunity (e.g., whole‐exome/whole‐genome sequencing) was not performed because it was not routinely indicated and was limited by resource and cost constraints.

Urgent contrast‐enhanced magnetic resonance imaging (CE‐MRI) of the abdomen demonstrated hepatosplenomegaly and a 7.0 × 6.7 cm multiseptated lobulated lesion involving the upper pole of the spleen with fluid–fluid levels and dependent contents showing diffusion restriction (ADC 0.9–1.0 × 10^−3^ mm^2^/s), consistent with infected contents suggestive of abscess/infected intracystic contents. The lesion exerted a mass effect on the gastric fundus and was associated with minimal surrounding perisplenic fat stranding. No intralesional gas was identified. Additional findings included a thin rim of left‐sided pleural effusion and mild bilateral perinephric fat stranding, more pronounced on the left (Figure 1). Following MRI Gastroenterology and surgical consultations were obtained. Endoscopic ultrasound (EUS) revealed a large, well‐defined hypoechoic collection in the superior pole of the spleen with internal septations and no calcifications. EUS‐guided fine needle aspiration was performed using a 22‐gauge needle after Doppler confirmation of vascular safety. Approximately 30 mL of purulent fluid was aspirated (Figure 2) and sent for cytopathology, GeneXpert MTB/RIF, mycobacterial culture, Gram staining and culture, acid‐fast bacilli smear, potassium hydroxide preparation, and fungal staining. Gram staining was performed, and the aspirated material was inoculated onto 5% sheep blood agar, chocolate agar, and MacConkey agar for aerobic bacterial culture, followed by incubation at 37°C. Growth of nonlactose fermenting colonies prompted subculture onto Xylose Lysine Deoxycholate (XLD) agar. Identification and antimicrobial susceptibility testing were performed using the VITEK 2 automated system (bioMérieux, France), which identified *Salmonella enterica* serovar Paratyphi A, susceptible to the antimicrobial agents tested. For fungal culture, the specimen was inoculated onto Sabouraud’s Dextrose Agar and incubated at both 25°C and 37°C. Creamy white yeast colonies appearing after 72 h at 37°C were identified as *Candida tropicalis* using the VITEK 2 system, with susceptibility to all antifungal agents tested. Immunostaining was negative for tubercular granuloma and malignancy. GeneXpert MTB/RIF, acid‐fast bacilli smear, tuberculosis PCR, and mycobacterial culture were all negative.

**FIGURE 1:**
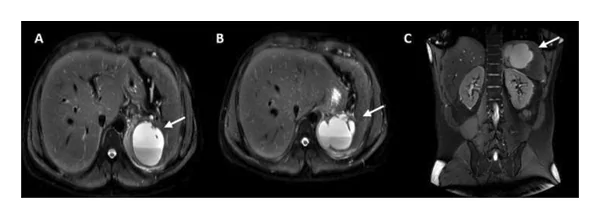
Contrast‐enhanced magnetic resonance imaging (MRI) of the abdomen demonstrating a multiseptated cystic lesion in the upper pole of the spleen with internal heterogeneous signal intensity and peripheral rim enhancement, suggestive of an infected splenic cyst/abscess. The lesion exerts a mass effect on the adjacent gastric fundus, with mild surrounding perisplenic fat stranding and a thin rim of left‐sided pleural effusion. Findings are consistent with a splenic abscess in the clinical context of fever and elevated inflammatory markers.

**FIGURE 2:**
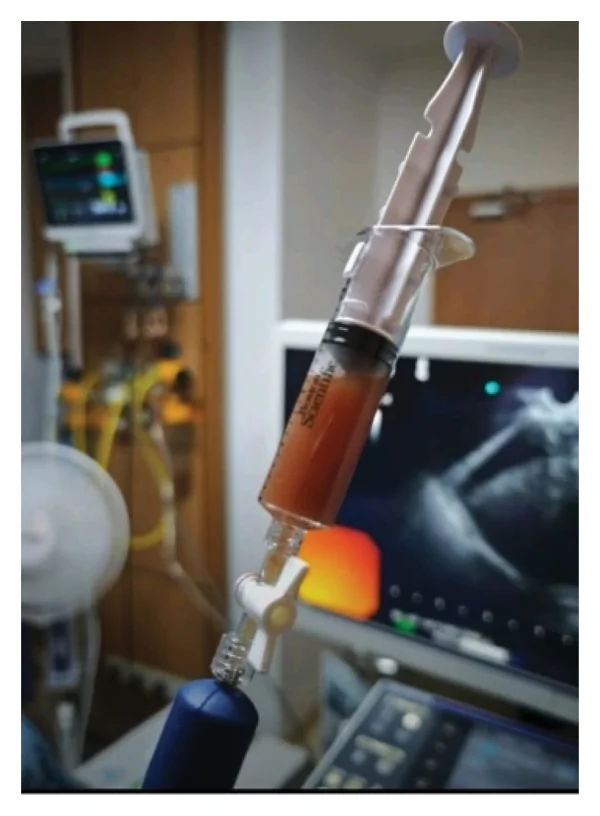
Purulent material obtained during splenic abscess drainage. Thick brownish purulent aspirate obtained during laparoscopic drainage and debridement of the splenic abscess. The presence of organized pus within the splenic cavity confirmed active suppurative infection and necessitated surgical source control.

Blood cultures were obtained before initiation of antimicrobial therapy at our centre; however, the patient had already received empirical antibiotics prior to referral, which may have reduced the diagnostic yield. The recovery of both organisms from purulent material aspirated from a radiologically confirmed splenic abscess, a normally sterile site, together with the compatible clinical presentation, laboratory and radiological findings, and subsequent clinical response to targeted antibacterial and antifungal therapy, strongly supports true polymicrobial infection rather than contamination. Semiquantitative colony counts and repeat cultures were not performed, in accordance with routine laboratory practice for sterile‐site aspirates.

## 3. Management and Follow‐Up

Prior to referral to our centre, the patient had received sequential empirical antibacterial therapy with oral cefuroxime, intravenous ceftriaxone, and subsequently intravenous meropenem because of persistent fever and abdominal pain. On admission to our centre, meropenem (1 g every 8 h) was continued, while intravenous teicoplanin (400 mg every 12 h for three loading doses, followed by 400 mg once daily) and oral doxycycline (100 mg twice daily) were added to provide broader empirical coverage while microbiological investigations were pending. EUS‐guided aspiration of the splenic abscess subsequently yielded purulent material for microbiological analysis. This culture of the splenic aspirate grew *Salmonella enterica* serovar Paratyphi A, which was susceptible to ceftriaxone, ampicillin, and trimethoprim‐sulfamethoxazole but resistant to ciprofloxacin, together with *Candida tropicalis*, which was susceptible to all antifungal agents tested (Table 1). Following availability of the susceptibility results and initial clinical improvement after drainage, broad‐spectrum empirical antibacterial therapy was discontinued, and treatment was de‐escalated to intravenous ceftriaxone (2 g twice daily). Oral trimethoprim‐sulfamethoxazole (double‐strength tablet twice daily) was added during the initial 10 days as additional targeted therapy against *Salmonella* but was subsequently discontinued because of significant gastrointestinal intolerance. Intravenous ceftriaxone was continued for a total of 21 days, followed by oral cefixime (400 mg twice daily) for an additional 2 weeks, completing a total of 5 weeks of targeted antibacterial therapy in conjunction with definitive source control, resulting in complete clinical, laboratory, and radiological recovery.

**TABLE 1:** Antimicrobial susceptibility profile of the *Salmonella enterica* serovar Paratyphi A and *Candida tropicalis* isolates recovered from splenic abscess aspirate.

Organism	Antimicrobial agent	MIC (µg/mL)	Interpretation
*Salmonella enterica* serovar Paratyphi A	Ceftriaxone	≤ 0.25	Sensitive
	Ciprofloxacin	2	Resistant
	Trimethoprim/Sulfamethoxazole	≤ 20	Sensitive
	Chloramphenicol^∗^	—	Sensitive
	Ampicillin^∗^	—	Sensitive

*Candida tropicalis*	Fluconazole	1	Sensitive
	Voriconazole	≤ 0.12	Sensitive
	Caspofungin	≤ 0.12	Sensitive
	Micafungin	≤ 0.06	Sensitive
	Amphotericin B	≤ 0.25	Sensitive

^∗^Chloramphenicol and ampicillin susceptibility for *Salmonella enterica* serovar Paratyphi A was determined by the disc diffusion method.

Empirical antifungal therapy was initially withheld because the pretest probability of invasive fungal infection in this immunocompetent patient was considered low. However, isolation of *Candida tropicalis* from purulent material obtained directly from the normally sterile splenic cavity, together with the clinical and radiological evidence of an abscess, strongly supported true fungal co‐infection rather than colonization or contamination. As the isolate was susceptible to all antifungal agents tested, intravenous fluconazole was initiated with an 800‐mg loading dose on Day 1, followed by 400 mg once daily for 7 days. Treatment was subsequently transitioned to oral fluconazole 400 mg once daily and continued to complete a total antifungal treatment duration of 6 weeks, guided by clinical and radiological resolution.

Despite targeted antimicrobial therapy, repeat contrast‐enhanced computed tomography (CECT) of the abdomen performed one week after admission demonstrated interval enlargement of the splenic abscess with persistent inflammatory changes. In view of radiological progression despite appropriate antimicrobial therapy, definitive source control was achieved by laparoscopic drainage and debridement of the splenic abscess. Intraoperatively, thick brownish purulent material was evacuated, necrotic and infected debris was carefully debrided, and the abscess cavity was irrigated until clear effluent was obtained (Figure 2). An intra‐abdominal drain was placed and removed after 5 days when drainage became minimal, and postoperative imaging confirmed no significant residual collection. Following surgical drainage, the patient became afebrile, his abdominal pain resolved completely, and inflammatory markers progressively normalized. He completed the planned courses of targeted antibacterial and antifungal therapy and was discharged in a stable clinical condition. At the two‐week outpatient follow‐up, ultrasonography of the abdomen demonstrated no residual or recurrent fluid collection. Serial ultrasonographic examinations performed over the subsequent six weeks showed progressive resolution without recurrence (Table 2). Fortnightly laboratory monitoring throughout therapy demonstrated normalization of inflammatory markers, with a CRP level of 6 mg/L, normal leukocyte counts, and other biochemical parameters.

**TABLE 2:** Timeline of clinical course and management.

Timeline	Clinical course and interventions
Day −10 to Day 0	Developed high‐grade fever, left upper quadrant abdominal pain, vomiting, and diarrhoea. Ultrasonography at the referring hospital demonstrated an infected splenic cyst/abscess. Empirical treatment was initiated with oral cefuroxime, followed by intravenous ceftriaxone. Because of persistent symptoms after 48 h, treatment was escalated to intravenous meropenem.
Day 0: Admission	Referred to our centre because of persistent fever and abdominal pain despite antimicrobial therapy. Meropenem was continued, and empirical coverage was broadened with teicoplanin and doxycycline. Laboratory investigations showed elevated inflammatory markers; blood cultures and evaluation for underlying immunodeficiency were negative.
Day 4	Contrast‐enhanced MRI demonstrated a multiseptated splenic abscess. Endoscopic ultrasound‐guided aspiration yielded approximately 30 mL of purulent material, which was submitted for bacterial, fungal, and mycobacterial investigations.
Days 5–8	Splenic aspirate culture grew *Salmonella enterica* serovar Paratyphi A and *Candida tropicalis*. Antibacterial therapy was de‐escalated to intravenous ceftriaxone according to susceptibility results, and fluconazole was initiated for fungal co‐infection. Trimethoprim‐sulfamethoxazole was added initially but subsequently discontinued because of gastrointestinal intolerance.
Approximately Day 10	Repeat contrast‐enhanced CT demonstrated interval enlargement of the splenic abscess despite targeted antimicrobial therapy, indicating inadequate source control.
Day 13	Laparoscopic drainage and debridement of the splenic abscess were performed. Thick purulent material and necrotic debris were evacuated, the cavity was irrigated, and an intra‐abdominal drain was placed.
Day 19	The drain was removed after drainage became minimal and postoperative imaging showed no significant residual collection. The patient was clinically stable and was discharged with continuation of oral therapy and outpatient follow‐up.
Follow‐up	Intravenous ceftriaxone was administered for a total of 21 days, followed by oral cefixime for an additional 2 weeks, completing 5 weeks of antibacterial therapy. Fluconazole was continued for a total of 6 weeks. Serial ultrasonography demonstrated progressive resolution without recurrence.

## 4. Discussion

Splenic abscess is an uncommon but clinically significant condition associated with considerable morbidity and mortality if diagnosis is delayed. Historically, it has been strongly linked to immunocompromised states such as diabetes mellitus, hematological malignancy, infective endocarditis, pancreatic disease, and posttransplant immunosuppression [4–7]. However, recent series suggest that splenic abscess can also occur in immunocompetent individuals, particularly in the presence of pre‐existing structural abnormalities such as splenic cysts [8–10]. The present case is noteworthy for several reasons. First, it occurred in a young immunocompetent adult without identifiable systemic risk factors. Second, it developed in the setting of an infected splenic cyst/abscess, supporting the concept that structural splenic lesions may act as a nidus for secondary hematogenous seeding. Third, microbiological analysis revealed dual infection with *Salmonella Paratyphi A* and *Candida tropicalis*, an exceptionally rare combination.

The microbiological spectrum of splenic abscess is diverse, with Gram‐negative bacilli, *Staphylococcus aureus*, and streptococci representing the most frequently isolated pathogens [3, 11, 12]. *Salmonella* species account for a small proportion of cases and are reported predominantly in endemic regions or among immunocompromised individuals. In contrast, fungal splenic abscesses, particularly those caused by *Candida* species, are typically encountered in patients with hematological malignancies, prolonged neutropenia, solid organ transplantation, disseminated candidemia, or other forms of severe immunosuppression [11, 13]. Coinfection involving bacterial and fungal pathogens of the spleen remains exceedingly rare, and published data remain limited to isolated reports.

*Salmonella*‐associated splenic abscess represents a rare extraintestinal manifestation of enteric fever. Most reported cases, including those from India, have been monomicrobial and successfully managed with prolonged antimicrobial therapy combined with image‐guided drainage or splenectomy, depending on abscess size, complexity, and clinical response [14–16]. To our knowledge, concurrent isolation of *Salmonella enterica* serovar Paratyphi A and *Candida tropicalis* from a splenic abscess has not been previously reported, highlighting the novelty of this case. Although *Candida tropicalis* splenic infection is typically encountered in patients with significant immunosuppression or healthcare‐associated risk factors, none of the established predisposing conditions were present in our patient [11]. He had no diabetes mellitus, HIV infection, malignancy, neutropenia, organ transplantation, chronic corticosteroid exposure, central venous catheter, prosthetic device, recent abdominal surgery, or other identifiable epidemiological risk factors for invasive candidiasis. We postulate that hematogenous seeding of a pre‐existing splenic cyst by *Salmonella* initiated the infectious process, while intracystic stasis, necrotic debris, and prior exposure to broad‐spectrum antibiotics created a favorable microenvironment for secondary fungal proliferation. Although the precise source of *Candida tropicalis* could not be established, its isolation from purulent material aspirated from a normally sterile splenic cavity, together with the compatible clinical presentation, radiological findings, and response to culture‐directed antibacterial and antifungal therapy, strongly supports true polymicrobial infection rather than contamination. Consistent with the current emphasis on spleen‐preserving management whenever clinically feasible, laparoscopic drainage provided definitive source control while preserving splenic function. This approach successfully avoided splenectomy and its associated lifelong risk of overwhelming postsplenectomy infection, underscoring the value of minimally invasive spleen‐conserving strategies in selected patients.

Our case expands the existing literature by demonstrating that such dual infection may occur even in an immunocompetent host. Clinically, splenic abscess commonly presents with nonspecific manifestations, particularly fever and left upper quadrant abdominal pain, which may contribute to delayed diagnosis. Cross‐sectional imaging, especially CECT, remains central to early detection and characterization of abscess morphology. Management strategies have shifted over recent decades toward splenic preservation. Image‐guided percutaneous drainage combined with targeted antimicrobial therapy is increasingly considered first‐line treatment [11]. However, failure of conservative therapy, enlarging collections, or complex multiloculated abscesses may necessitate surgical drainage or splenectomy [11, 12]. In this case, radiological progression despite appropriate antimicrobial therapy required laparoscopic drainage and debridement, resulting in rapid clinical improvement and preservation of splenic function. The optimal duration of antimicrobial therapy for splenic abscess is not clearly established and should be individualized according to the causative organism, adequacy of source control, clinical response, and radiological resolution. Our patient completed a total of 5 weeks of culture‐directed antibacterial therapy and 6 weeks of antifungal therapy, together with definitive source control, resulting in complete clinical and radiological resolution. This case underscores the importance of comprehensive microbiological evaluation including fungal studies in patients with splenic abscess, even when immunocompetent. Early source control remains critical to achieving favorable outcomes and minimizing the need for splenectomy.

## 5. Conclusion

Dual bacterial‐fungal splenic abscess is rare, particularly in immunocompetent individuals. This case highlights the potential for polymicrobial infection in the setting of an infected splenic cyst and emphasizes the importance of early imaging, comprehensive microbiological workup, and timely source control. A multidisciplinary, organ‐preserving approach combining targeted antimicrobial therapy and minimally invasive drainage can lead to complete recovery while preserving splenic immune function.

## Author Contributions

Kapil Chahal, Neha Rastogi, Swati Bhayana, Vikas Dua, and Rahul Bhargava contributed to drafting the manuscript. Kapil Chahal, Neha Rastogi, Amit Javed, Rinkesh Kumar Bansal, Zubin Dev Sharma, Saurabh Kumar, and Narola Yanger were involved in the clinical management of the patient.

## Funding

No funding was obtained for this study.

## Disclosure

All authors reviewed and approved the final manuscript.

## Ethics Statement

Ethical approval was not required for this study, as it is a single case report describing routine clinical management. Written informed consent was obtained from the patient for publication of this case report.

## Conflicts of Interest

The authors declare no conflicts of interest.

## Data Availability

This manuscript does not report data generation or analysis.
